# Premenstrual Syndrome-Impact Questionnaire: Cross-cultural adaptation, reliability, and validity of the Turkish version

**DOI:** 10.1590/1516-3180.2024.0288.R1.07042025

**Published:** 2025-09-15

**Authors:** Halime Arıkan, Erkan Erol

**Affiliations:** I Physiotherapist, Assistant Professor, Department of Physiotherapy and Rehabilitation, Faculty of Health Sciences, Tokat Gaziosmanpaşa University, Tokat, Türkiye.; II Physiotherapist, Assistant Professor, Department of Physiotherapy and Rehabilitation, Faculty of Health Sciences, Tokat Gaziosmanpaşa University, Tokat, Türkiye.

**Keywords:** Premenstrual dysphoric disorder, Premenstrual syndrome, Validation study [publication type], Reproducibility of results, Data management, Premenstrual Syndrome Impact Questionnaire, Reliability, Validity, Turkish version

## Abstract

**BACKGROUND:**

Short and practical questionnaires and tests that assess premenstrual symptoms and premenstrual syndrome (PMS) are required.

**OBJECTIVES:**

This study aimed to investigate the cross-cultural adaptation, validity, and reliability of the Premenstrual Syndrome Impact Questionnaire (PMS-IQ) in Turkish women with PMS.

**DESIGN AND SETTING:**

The reliability and validity of the questionnaire were evaluated in Tokat, Türkiye.

**METHODS:**

A convenience sample of 146 individuals diagnosed with PMS was used to assess the reliability and validity of the Turkish version of the PMS-IQ. Test-retest analyses were performed in a subset of 96 individuals one week after the initial assessment. Construct validity was evaluated through convergent validity analysis using the Premenstrual Syndrome Scale (PMSS) and the Premenstrual Symptoms Impact Survey (PMSIS), and divergent validity analysis with the Big Five Inventory-10 (BFI-10).

**RESULTS:**

Cronbach’s **α** values for the total score and subscales ranged from 0.861 to 0.917, whereas the test-retest reliability values ranged from 0.755 to 0.847. Factor analysis indicated that the scale had a three-factor structure. The total PMS-IQ score was significantly correlated with both the PMSS (r = 0.718) and PMSIS (r = 0.774), but showed no significant correlation with the BFI-10 (r = 0.113). No floor or ceiling effects were observed for the total or subscale scores of the PMS-IQ.

**CONCLUSIONS:**

The Turkish version of the PMS-IQ demonstrated reliability and validity for evaluating individuals with PMS.

**CLINICAL TRIAL REGISTRATION:**

This study is registered at ClinicalTrials.gov (identifier: NCT05725447).

## INTRODUCTION

 Menstruation is a physiological process that recurs monthly for approximately 30–35 years, significantly affecting women’s lives.^1^ Women in Turkey commonly experience premenstrual syndrome (PMS).^ 2,3^ PMS is characterized by a cluster of physical, behavioral, and emotional symptoms that manifest during the luteal phase and subside with the onset of menstruation or shortly thereafter.^4^ Although the exact causes of PMS remain unclear, it is believed to be associated with hormonal changes, neurotransmitters, prostaglandins, dietary habits, medications use, and lifestyle factors.^5^ Approximately 90% of women of reproductive age group report experiencing premenstrual symptoms of varying severity.^4^ Emotional and mood-related symptoms frequently observed in PMS include mood swings, depression, anger, irritability, changes in sadness, tension, heightened sensitivity, and crying. Physical symptoms may include weight gain, abdominal cramps, acne, fatigue, breast tenderness, and bloating.^6^

 Considering individual and psychological differences, it is important to manage mild to severe PMS symptoms during each menstrual cycle. Occasionally, the symptoms become severe enough to adversely influence daily activities. PMS is also associated with significant psychological issues.^7,8^ Studies conducted among students have shown that those experiencing moderate to severe PMS symptoms report difficulties in concentration, acute psychological distress, decreased academic achievement, and negative thoughts.^9,10^ Various interventions, including relaxation techniques, have been investigated for the management of PMS.^11^ If left untreated, severe PMS symptoms may worsen mental status in response to personal and environmental stressors, potentially leading to the development of premenstrual dysphoric disorder (PMDD), a more severe form of PMS.^12,13^

 Most PMS questionnaires measure the presence and/or severity of past and future symptoms rather than the impact of those symptoms.^14-16^ The Premenstrual Syndrome Impact Questionnaire (PMS-IQ), comprising 18 items, is designed to evaluate functional interactions and psychological stress associated with premenstrual symptoms in daily life. The PMS-IQ evaluates the impact of the complex and multifaceted nature of PMS, facilitates the diagnostic process, and enables the planning and evaluation of treatment.^17^

## OBJECTIVES

 Questionnaires and tests that assess premenstrual symptoms and PMS are required. Broadening the range of assessment approaches would enable more comprehensive evaluation of the disorder. This cross-cultural adaptation study aimed to validate a self-administered Turkish version of the PMS-IQ (PMSIQ/T). 

## METHODS

### Individuals

 The study sample comprised women aged 18–45 years with PMS, including students and employees of Tokat Gaziosmanpaşa University. All participants were informed of the study’s aim and methodology, which were approved by the Ethics Committee of Tokat Gaziosmanpaşa University (decision date: November 3, 2022; decision no: 83116987-760). Eligibility required meeting the provisional diagnostic criteria for PMDD as defined in the Diagnostic and Statistical Manual of Mental Disorders (DSM), 5th Edition,^18^ confirmed through a prospective screening process. 

 The exclusion criteria were as follows: (1) presence of bipolar disorder, psychosis, moderate to severe depression, eating disorder, or somatic symptom disorders; (2) current or previous participation in a physiotherapy program due to PMS symptoms; (3) immediate suicidal inclinations; (4) pregnancy, childbirth, or breastfeeding within the last 3 months; (5) gynecological conditions such as infertility, hysterectomy, endometriosis, oophorectomy, polycystic ovarian syndrome, or gynecological cancer; and (6) initiation or changes in the use of contraceptive tablets, antidepressants, hormones (e.g., thyroid hormones), or benzodiazepines/antipsychotics within the last 3 months. 

 The required sample size was calculated as 99, based on an expected intraclass correlation coefficient (ICC) of ρ1 = 0.85,^19^ a minimal acceptable reliability level of ρ0 = 0.75,^13^ with α = 0.05 and β = 0.20. 

**Figure 1:**
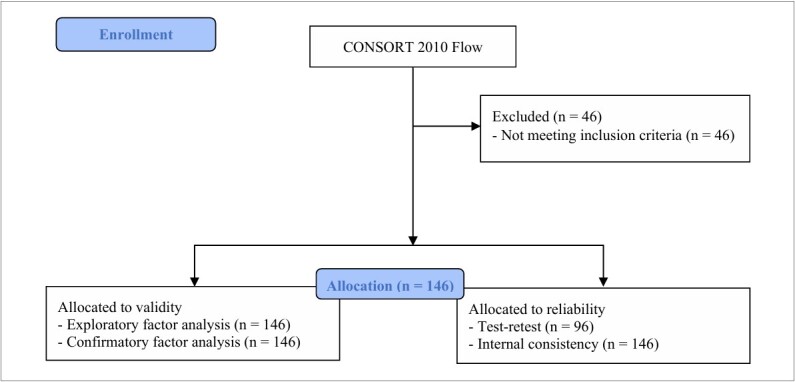
Flowchart of participant recruitment.

 A total of 192 women were initially recruited. Forty-six were excluded due to either gynecological diseases (n = 15) or failure to meet PMDD criteria (n = 31). Thus, the final sample included 146 participants (**Figure 1**). Before the study began, all participants were informed about the study, voluntarily agreed to participate in accordance with the principles of the Helsinki Declaration, and formally signed an informed consent form. 

### Procedure

 Before commencing the study, approval was obtained from J. N. Kues,^17^ the developer of the PMS-IQ. The language translation and cultural adaptation process of the PMS-IQ followed the methodology outlined by Beaton.^20^ The original questionnaire was independently translated into Turkish by two individuals (a physiotherapist who was unaware of the study and an English linguist). These translators than collaborated to merge their translations into a single version. Subsequently, two certified translators independently performed a back-translation of the merged Turkish version into English. The version translated back into English from Turkish was reviewed by a translation team (a physiotherapist, an English linguist, and two certified translators), who evaluated its compatibility with the original questionnaire. A clarity form was developed for each question in the questionnaire and piloted. During the pretest phase, 146 individuals with PMS were assessed for their comprehension of the items and wording, as well as their ability to complete the questionnaire.^20^ Comprehensibility of the questionnaire was evaluated using a binary scoring system, with responses recorded as either "yes" or "no". If a participant responded "no" for comprehensibility, they were asked to identify the unclear items and explain the reason. Based on the results of the pretest phase, no modifications were made to the pre-final version of the PMS-IQ/T. 

### Measures

 Premenstrual Syndrome Scale (PMSS): The PMSS was developed by Gençdoğan in 2006 to access the severity of premenstrual symptoms based on DSM-III and DSM-IV-R criteria. Widely used in Turkey, the scale consist of 44 items, each referring to experiences "one week before menstruation". It covers nine dimensions: depressive affect, anxiety, fatigue, irritability, depressive thoughts, pain, appetite changes, sleep changes, and bloating. Each item is rated on a five-point Likert scale ranging from 1 to 5. The PMSS total score ranges from 44 to 220 points. Dimensional scores are calculated by summing the items within each dimension, and the overall PMSS score is derived by summing the scores of each dimension. Individuals with a total score exceeding 50% are categorized as PMS-positive. A high PMSS score is indicative of intense premenstrual symptoms.^21^

 Premenstrual Symptoms Impact Survey (PMSIS): Wallenstein et al. developed the PMSIS, a six-item scale designed to assess the influence of premenstrual symptoms on health-related quality of life. The scale consists of six items that assess the impact of premenstrual symptoms on various aspects of quality of life, including cognitive well-being, interpersonal interaction, liveliness, and responsibility management. Each is rated on a five-point scale, from 1 (no impact) to 5 (high impact), reflecting the severity of the impact on various aspects of quality of life. Total scores range from 6 to 30, with higher scores indicating a decrease in the quality of life associated with the impact of premenstrual symptoms.^16^ Güler et al. conducted a validity and reliability study of the Turkish version of the PMSIS. The scale is proficient in evaluating the condition and treatment outcomes of PMS in women of reproductive age.^22^

 Big Five Inventory-10 (BFI-10): The BFI-10 was introduced by Rammstedt and John as a concise alternative to the longer BFI44.^23^ The scale comprises 10 items organized into five sub-dimensions. Responses are rated on a 5-point response scale: "disagree strongly," "disagree a little," "neither agree nor disagree," "agree a little," and "agree strongly." Statements 1, 3, 4, 5, and 7 are inverted on the scale. The Turkish version of the BFI-10 has been validated and shown to be reliable.^24^

### Statistical Analysis

 Statistical analyses were performed using SPSS version 22.0 (IBM Corp., Armonk, NY, USA) and LISREL version 8.80 (Scientific Software International, Inc., Lincolnwood, IL, USA). Data are presented as mean ± standard deviation, median (minimummaximum), and percentage (%). The Kolmogorov–Smirnov test was used to determine whether the numerical variables were normally distributed. 

 Internal consistency and test-retest reliability analyses were conducted to assess the reliability of the PMS-IQ. Cronbach’s α was employed for internal consistency analysis, whereas the ICC with a 95% confidence interval was used for the test-retest analysis. A Cronbach’s α value ≥ 0.80 is considered an acceptable threshold,^25^ whereas an ICC score ≥ 0.75 is considered acceptable for test-retest reliability.^26^

 Reproducibility was examined using the minimum detectable change (MDC) and the standard error measurement (SEM). The following formulae were applied:^27^ ${\mathrm{MDC}}_{95}=z\times \mathrm{SEM}\times \sqrt{2}$ ${\mathrm{SEM}}_{95}=\mathrm{SD}\times \sqrt{1-\mathrm{ICC}}$

 Exploratory factor analysis (EFA) was conducted to assess the structural validity of the PMS-IQ. Before factor analysis, sample suitability was evaluated using Bartlett’s test, and sample adequacy was assessed using the Kaiser–Meyer Olkin test.^28^ Confirmatory factor analysis (CFA) was performed to validate and confirm the factor structure identified in the initial analysis. The fit indices supporting this analysis were also examined.^29^

 Convergent and divergent validity were assessed for construct validity using Pearson’s and Spearman’s correlation analyses. The convergent and divergent validity of the PMS-IQ/T was determined based on the total and subscale scores obtained from the PMS-IQ, PMSS, PMSIS, and BFI-10. Correlation coefficients were interpreted according to established thresholds in medical research: 0.90-1.00 (very high), 0.70-0.90 (high), 0.50-0.70 (moderate), 0.30-0.50 (low), and 0.00-0.30 (negligible).^30^

 The percentages of the lowest and highest values for the PMSIQ/T and its subscales were calculated to assess ceiling and floor effects.^31^ P < 0.05 was considered statistically significant. 

## RESULTS

**Table 1** outlines the characteristics of the participants. A total of 146 Turkish-speaking females with PMS completed the questionnaire, of whom 96 completed it at the second time point. 

**Table 1:** Numerical and categorical characteristics of participants in the test and retest groups

**Numeric variables**		**Mean (SD) (test) (n = 146)**	**Mean (SD) (retest) (n = 96)**
Age (years)		20.69 (1.96)	20.94 (2.18)
Menarche age (years)		13.15 (1.31)	13.28 (1.29)
Weight (kg)		58.06 (9.86)	57.68 (10.32)
Height (cm)		163.64 (6.10)	163.72 (6.04)
BMI (kg/m^2^)		21.73 (3.33)	21.61 (3.58)
Categorical variables		n (%)	n (%)
Menstrual cycle			
	20 days and less	8 (5.5 %)	4 (4.2 %)
	21-33 days	122 (83.6 %)	82 (85.4 %)
	34 days and more	16 (10.9 %)	10 (10.4 %)
Menstrual order			
	Yes	115 (78.8 %)	72 (76.6 %)
	No	31 (21.2 %)	22 (23.4 %)
Premenstrual drug use			
	Yes	45 (30.8 %)	31 (32.3 %)
	No	101 (69.2 %)	65 (67.7 %)
Menstrual drug use			
	Yes	55 (37.7 %)	40 (41.7 %)
	No	37 (25.3 %)	21 (21.9 %)
	Sometimes	54 (37.0 %)	35 (36.4 %)
Smoking			
	Yes	23 (15.8 %)	14 (14.6 %)
	No	123 (84.2 %)	82 (85.4 %)
Use of alcohol			
	Yes	5 (3.4 %)	5 (5.2 %)
	No	141 (96.6 %)	91 (94.8 %)
Painful menstruation			
	No	4 (2.7 %)	3 (3.1 %)
	Mild	18 (12.3 %)	11 (11.5 %)
	Moderate	41 (28.1 %)	24 (25.0 %)
	Severe	63 (43.2 %)	44 (45.8 %)
	Seriously	20 (13.7 %)	14 (14. 6%)

SD = standard deviation; BMI = body mass index.

 Internal consistency, as measured by Cronbach’s α, was excellent (α = 0.917). Test–retest reliability of the PMS-IQ/T was also very high (ICC = 0.847; 95% CI = 0.780-0.895). The SEM for the total score was 3.79, and the MDC was 10.50. Cronbach’s α, ICC, SEM, and MDC values for the total and subscale scores are presented in **Table 2**. 

**Table 2:** Test–retest reliability and internal consistency of the Turkish version of the Premenstrual Syndrome Impact Questionnaire (n = 96)

	**Baseline Mean ± SD**	**Retest Mean ± SD**	**P**	**Test – retest (ICC and 95% CI)**	**SEM**	**MDC**	**Internal consistency (Cronbach’s α)**
PMS-IQ/T total score	40.54 ± 9.84	40.46 ± 9.54	0.879	0.847 (0.780-0.895)	3.79	10.50	0.917
Factor 1	19.65 ± 6.15	19.68 ± 5.91	0.932	0.824 (0.747-0.879)	2.53	7.02	0.903
Factor 2	9.02 ± 2.53	9.13 ± 2.49	0.561	0.758 (0.657-0.831)	1.24	3.43	0.862
Factor 3	11.76 ± 2.45	11.45 ± 2.32	0.070	0.755 (0.654-0.830)	1.18	3.27	0.861

PMS-IQ/T = Turkish version of the Premenstrual Syndrome Impact Questionnaire; SD = standard deviation; ICC = intraclass correlation coefficient; CI = confidence intervals; SEM = standard error measurement; MDC = minimal detectable change.

 The mean scores of the PMS-IQ/T, corrected item-total correlations, and Cronbach’s α for item deletion are presented in **Table 3**. 

**Table 3:** Mean scores, corrected item-total correlations, and Cronbach’s α if item deleted for the Turkish version of the Premenstrual Syndrome Impact Questionnaire (n = 146)

**Item**	**Mean**	**SD**	**Corrected item total correlation**	**Cronbach’s α if item deleted**
1	2.47	0.78	0.567	0.913
2	2.76	0.79	0.489	0.914
3	2.66	0.89	0.501	0.914
4	1.57	0.70	0.456	0.915
5	2.34	0.88	0.505	0.914
6	1.75	0.98	0.324	0.919
7	1.83	0.88	0.648	0.910
8	2.10	1.03	0.677	0.909
9	2.30	0.87	0.671	0.910
10	1.75	0.85	0.614	0.911
11	2.43	0.95	0.551	0.913
12	2.01	0.95	0.531	0.913
13	2.50	0.99	0.736	0.908
14	2.62	0.96	0.627	0.911
15	2.33	0.96	0.684	0.909
16	2.14	0.94	0.675	0.910
17	2.39	0.93	0.590	0.912
18	2.39	1.03	0.735	0.908

SD = standard deviation.

**Figure 2:**
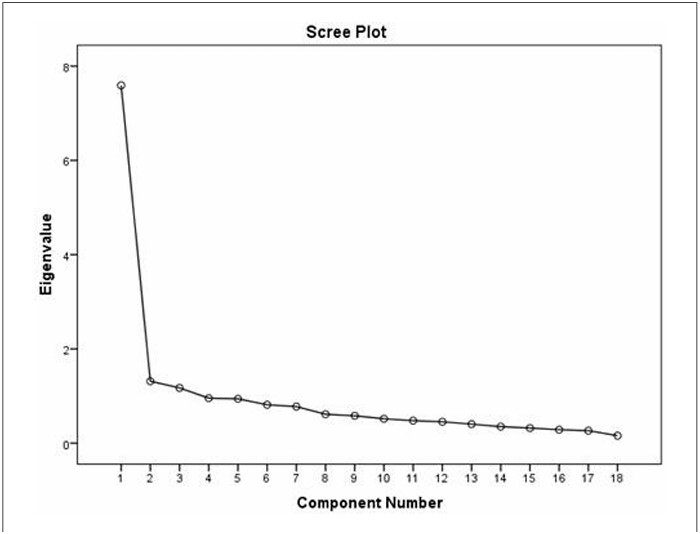
Scree plot of the Turkish Version of the Premenstrual Syndrome Impact Questionnaire (n = 146).

 The Kaiser–Meyer–Olkin (KMO) test confirmed the adequacy of the sample for analysis (KMO = 0.884). The correlations between the items of the PMS-IQ/T were deemed sufficient for analysis, as evidenced by Bartlett’s sphericity test (chi-squared = 1219.442, P < 0.001). After removing factors with eigenvalues > 1, the 18 items of the PMS-IQ/T were associated with three factors. **Figure 2** shows the distribution of the eigenvalues through a scree plot. This factor structure explained 55.996% of the total variance, which is considered satisfactory as it accounts for > 50% of the total variance in the PMS-IQ/T. According to the EFA results, all factor loadings were above 0.40 (**Table 4**). 

**Table 4:** Factor analysis results for the Turkish version of the Premenstrual Syndrome Impact Questionnaire (n = 146)

**Item**	**Factor 1**	**Factor 2**	**Factor 3**
7	0.653		
8	0.711		
10	0.704		
11	0.588		
12	0.637		
13	0.641		
15	0.695		
16	0.669		
18	0.691		
6		0.436	
9		0.515	
14		0.795	
17		0.752	
1			0.570
2			0.564
3			0.715
4			0.545
5			0.695

Kaiser-Meyer-Olkin Measure of Sampling Adequacy = 0.884; Bartlett’s Test of Sphericity = 1219.442; Total variance = 55.996%

 The three factors observed in the EFA were confirmed using CFA. The comparative Fit Index, Tucker-Lewis Index, Incremental Fit Index, Chi-square/Degrees of freedom, Consistent Akaike Information Criterion, Expected Cross-Validation Index, and Root Mean Square Error of Approximation were 0.95, 0.95, 0.95, 2.11, 511.36, 2.46, and 0.087, respectively, according to the CFA results. These values are considered excellent and acceptable.^29^

 Convergent validity analysis revealed that the PMS-IQ/T was significantly correlated with the PMSS and PMSIS (**Table 5**). Divergent validity results indicated a non-significant correlation with the BFI-10. Recognizing the established association between neuroticism and premenstrual symptoms related to anxiety,^32^ the original study predicted a low but noteworthy correlation with the neuroticism scale.^17^

**Table 5:** Correlations between the Turkish version of the Premenstrual Syndrome Impact Questionnaire and its subscales with other questionnaires for convergent and divergent validity (n = 146)

	**PMS-IQ total**	**Factor 1 (psychological impact)**	**Factor 2 (recreational and emotional impact)**	**Factor 3 (motivational impact)**
Convergent validity				
PMSS	0.718^**^ μ	0.712^**^	0.480^**^ μ	0.593^**^ μ
-Depressive feelings	0.597^**^ μ	0.634^**^	0.316^**^ μ	0.529^*^ μ
-Anxiety	0.568^**^	0.612^**^	0.338^**^	0.397^**^
-Fatigue	0.569^**^	0.530^**^	0.450^**^	0.529^**^
-Irritability	0.571^**^	0.552^**^	0.365^**^	0.512^**^
-Depressive thinking	0.701^**^ μ	0.695^**^	0.483^**^ μ	0.539^**^ μ
-Pain	0.524^**^	0.491^**^	0.452^**^	0.370^**^
-Changed appetite	0.108	0.138	0.022	0.096
-Changed sleep	0.458^**^	0.433^**^	0.329^**^	0.393^**^
-Swelling	0.293^**^	0.300^**^	0.223^**^	0.246^**^
PMSIS	0.774^**^	0.741^**^	0.591^**^	0.586^**^
Discriminant validity				
BFI-10	0.113μ	0.130	0.129μ	0.089μ
-Extraversion	-0.140μ	-0.139	-0.083μ	-0.079μ
-Agreeableness	-0.063μ	-0.121	0.013μ	-0.032μ
-Conscientiousness	0.042μ	0.073	-0.029μ	-0.025μ
-Neuroticism	0.300^**^ μ	0.284^**^	0.165^*^ μ	0.294^**^ μ
-Openness	0.199^*^ μ	0.195^*^	0.251^**^ μ	0.103μ

PMS-IQ = Premenstrual Syndrome Impact Questionnaire; PMSS = Premenstrual Syndrome Scale; PMSIS = Premenstrual Symptoms Impact Survey; BFI-10 = Big Five Inventory-10.

μ Pearson correlation analysis.

 The floor effect percentages were 0.7%, 2.7%, 5.5%, and 0.7% for the total score and factors 1, 2, and 3, respectively. The ceiling effects percentages were 0%, 0.7%, 1.4%, and 0.7%, respectively. 

 No ceiling or floor effects were observed in the PMS-IQ/T or its subscales, as all values were < 15%. 

## DISCUSSION

 This study investigated the validity and reliability of the PMSIQ/T in Turkish women diagnosed with PMS. The PMS-IQ/T demonstrated superior reliability and strong validity in evaluating functional interference and psychological distress. 

 Many instruments and screening tools have been developed to evaluate PMS. These include the Daily Record of Severity of Problems, Premenstrual Screening Tool (PSST), Premenstrual Record of Impact and Severity of Menstruation, Calendar of Premenstrual Experiences, Daily Symptom Report,^33^ Premenstrual Assessment Form (PAF),^34^ Premenstrual Coping Measure (PCM),^35^ PMSS,^21^ and PMSIS.^16^ The validity and reliability of the PSST,^18^ PAF,^36^ PCM,^37^ PMSS,^21^ and PMSIS^22^ have been examined in Turkish population. The Cronbach’s α coefficient of the Turkish version of the PSST was 0.92.^18^ The internal consistency of the PAF yielded a Cronbach’s α of 0.97.^36^ For the PCM subscores, Cronbach’s α values ranged from 0.751 to 0.890, and ICC ranged from 0.712 to 0.734.^37^ The PMSS had a Cronbach’s α of 0.75 and an ICC of 0.87.^21^ The PMSIS showed a Cronbach’s α of 0.89.^22^ In the original version of the PMS-IQ, the subscales had a Cronbach’s α of 0.90.^17^ In the present study, the PMS-IQ/T demonstrated Cronbach’s α values ranging from 0.861-0.917 and ICCs from 0.755-0.847 for the total score and subscales. These findings are consistent with previous literature and confirm the excellent reliability of the PMS-IQ/T. The Bland-Altman plots further supported these results. 

 The SEM is a reliability measure that evaluates the stability of responses across multiple measurements. It represents amount of error attributable to measurement variability. The MDC refers to the smallest perceptible and significant change in an evaluated parameter, indicating a change not due to measurement error. Both measurements are considered indicators of reliability.^26^ The SEM value was 3.79 points, corresponding to 9.36% of the mean PMSIQ/T score and 5.26% of the maximum score. The MDC value, calculated based on the SEM, was 10.50 points, which equates to 25.93% of the average value. Given the maximum score of 72 points, 10.50 points represents 14.58% of the maximum value. 

 When the corrected item-total correlation values of the PMSIQ/T were examined, moderate to high correlations were observed, indicating generally high correlations. The Cronbach’s α values for item deletion supported the inclusion of all 18 items in the PMSIQ/T. Removing any item did not result in a Cronbach’s α value higher than that of the total score (0.917), except for one item. For item 6, this value (0.919) was very close to the overall Cronbach’s α . Therefore, all items were included in the PMS-IQ/T. 

 The original version of the PMS-IQ exhibited a two-factor structure, as confirmed by both EFA and CFA. Following EFA, the total variance was 52.11%. The sub-dimensions were named "psychological impact" and "functional impact".^17^ Both the EFA and scree plot for the PMS-IQ/T revealed a three-factor structure, with each factor having an eigenvalue greater than one. The variance percentage for three factors obtained through the EFA was 55.996%, which is considered acceptable. As a result, PMS-IQ/T was segmented into three distinct subscales: "psychological impact," "recreational and emotional impact," and "motivational impact." 

 Divergent and convergent validity of the original PMS-IQ were assessed using the BFI-10 and PDI, with results indicating notable positive correlations ranging from low to high.^17^ The PMS-IQ/T demonstrated both convergent and divergent validity, supported by significant correlations between the PMS-IQ/T and its subscales with the PMSS, PMSIS, and BFI-10. A strong positive correlation was observed between the PMS-IQ/T and both the PMSS and PMSIS, whereas a weaker correlation was found with the BFI10. The divergent validity results using the BFI-10 were consistent with those of the original study. Moderate and high correlations between the PMS-IQ/T and the total scores and subscales of the PMSS and PMSIS indicated good validity. 

 While extensive statistical analyses were conducted to evaluate the psychometric properties of the PMS-IQ/T, a limitation of this study is the absence of responsiveness assessment. Consequently, further studies are warranted to assess responsiveness by determining the minimum clinically important differences. Further studies should also examine the diagnostic accuracy of the PMS-IQ/T in predicting PMS. In addition, the sensitivity and specificity of the PMS-IQ/T should be investigated. 

## CONCLUSIONS

 Based on the outcomes of this study, the PMS-IQ/T emerged as a robust and internally consistent tool, demonstrating validity and reliability in the assessment of individuals with PMS. These findings suggest that the PMS-IQ/T is suitable for use in both clinical practice and research settings. 
